# Continuous Infusion of Angiotensin IV Protects against Acute Myocardial Infarction via the Inhibition of Inflammation and Autophagy

**DOI:** 10.1155/2021/2860488

**Published:** 2021-12-14

**Authors:** Wen-wu Bai, Hao Wang, Chun-hua Gao, Ke-yin Liu, Bing-xiu Guo, Fan Jiang, Ming-xiang Zhang, Chen Li, Wei-dong Qin

**Affiliations:** ^1^Department of Traditional Chinese Medicine, Qilu Hospital, Cheeloo College of Medicine, Shandong University, Jinan, China; ^2^Department of Critical Care Medicine, Qilu Hospital, Cheeloo College of Medicine, Shandong University, Jinan, China; ^3^Department of Nursing, Uygur Medical Hospital of Kashgar, Kashgar, Xinjiang Uygur Autonomous Region, China; ^4^State Key Laboratory of Biobased Material and Green Papermaking, Key Laboratory of Pulp & Paper Science and Technology of Shandong Province/Ministry of Education, Qilu University of Technology, Shandong Academy of Sciences, Jinan, China; ^5^Key Laboratory of Cardiovascular Proteomics of Shandong Province, Qilu Hospital of Shandong University, Jinan, China; ^6^Key Laboratory of Cardiovascular Remodeling and Function Research, Chinese Ministry of Education, Chinese Ministry of Health and Chinese Academy of Medical Sciences, Qilu Hospital, Cheeloo College of Medicine, Shandong University, Jinan, China; ^7^School of Basic Medical Sciences, Shandong University, Jinan, China

## Abstract

Acute myocardial infarction (AMI) is a major cause of morbidity and mortality worldwide. Angiotensin (Ang) IV possesses many biological properties that are not yet completely understood. Therefore, we investigated the function and mechanism of Ang IV in AMI in *in vivo* and *in vitro* conditions. AMI was performed by ligation of the left anterior descending coronary artery (LAD) in male C57 mice. Ang IV was continuously infused by a minipump 3 d before AMI for 33 d. The neonatal rat ventricular myocytes (NRVCs) were stimulated with Ang IV and cultured under hypoxic conditions. *In vivo*, Ang IV infusion significantly reduced the mortality after AMI. By the 7^th^ day after AMI, compared with the AMI group, Ang IV reduced the inflammatory cytokine expression. Moreover, terminal deoxyribonucleotidyl transferase- (TDT-) mediated dUTP nick-end labeling (TUNEL) assay showed that Ang IV infusion reduced AMI-induced cardiomyocyte apoptosis. Compared with AMI, Ang IV reduced autophagosomes in cardiomyocytes and improved mitochondrial swelling and disarrangement, as assessed by transmission electron microscopy. By 30^th^ day after AMI, Ang IV significantly reduced the ratio of heart weight to body weight. Echocardiography showed that Ang IV improved impaired cardiac function. Hematoxylin and eosin (H&E) and Masson staining showed that Ang IV infusion reduced the infarction size and myocardial fibrosis. *In vitro*, dihydroethidium (DHE) staining and comet assay showed that, compared with the hypoxia group, Ang IV reduced oxidative stress and DNA damage. Enzyme-linked immunosorbent assay (ELISA) showed that Ang IV reduced hypoxia-induced secretion of the tumor necrosis factor- (TNF-) ɑ and interleukin- (IL-) 1*β*. In addition, compared with the hypoxia group, Ang IV reduced the transformation of light chain 3- (LC3-) I to LC3-II but increased p62 expression and decreased cardiomyocyte apoptosis. Overall, the present study showed that Ang IV reduced the inflammatory response, autophagy, and fibrosis after AMI, leading to reduced infarction size and improved cardiac function. Therefore, administration of Ang IV may be a feasible strategy for the treatment of AMI.

## 1. Introduction

Acute myocardial infarction (AMI) is the most severe manifestation of the coronary artery disease (CAD) [1], which causes more than a third of deaths in developed nations annually and remains the leading cause of death worldwide [2]. AMI results in myocardial remodeling and heart failure [3]. Apoptosis and necrosis of cardiomyocytes in response to infarction trigger an inflammatory response and an imbalance of collagen synthesis and degradation [4]. After AMI, myofibroblasts in the infarcted heart migrate into the injured myocardium, replace the damaged cardiomyocytes, and form scar tissue to avoid cardiac rupture. However, excessive myofibroblast accumulation and fibrosis in the uninjured area of the heart contribute to cardiac fibrosis and heart failure [5].

Autophagy is a type of cell death that is closely associated with the development of heart failure [6]. It is a process in which the cytoplasmic constituents are sequestered in double-membraned autophagosomes and then delivered to lysosomes for degradation [7]. Under physiological conditions, autophagy plays a critical role in maintaining the cardiomyocyte function and survival by removing the damaged organelles and protein aggregates [8]. However, dysregulated or excessive autophagy can contribute to the development of many cardiovascular diseases [9]. Moreover, autophagy induced by ischemia may play a protective or pathogenic role in heart disease [10]. Given the controversial role of autophagy during myocardial infarction (MI) and heart failure, a better understanding of the underlying molecular and cellular mechanisms is critical for preserving heart function after AMI.

Angiotensin [3–8], also known as angiotensin (Ang) IV, which is hydrolyzed from Ang II by dipeptidylaminopeptidase III or from Ang III by aminopeptidase N, has sparked great interest because of its wide range of physiological effects [11]. Ang IV mediates important physiological functions in the central nervous system, including blood flow regulation and processes attributed to learning and memory [12]. The antiapoptotic role of Ang IV has also been reported in an experimental study [13]. In addition, chronic Ang IV infusion improves endothelial function in early and advanced atheroma [14]. A medium dose of Ang IV reduces the inflammatory response, providing a novel approach for the treatment of abdominal aortic aneurysm [15]. However, the exact role and underlying mechanism of Ang IV in AMI remain unclear.

Therefore, in the present study, we aimed to investigate the role and underlying mechanism of Ang IV in AMI and cardiac remodeling by ligating the left anterior descending coronary artery (LAD) in mice and culturing neonatal rat ventricular myocytes (NRVCs) under hypoxic conditions. We aimed to identify a novel target for the treatment of AMI.

## 2. Materials and Methods

### 2.1. Animals

The animal experiments were approved by the Animal Care and Use Committee of Shandong University (Jinan, Shandong, China). C57 male mice (10 weeks old; Charles Rivers, Beijing, China) were housed in a pathogen-free animal care facility on a 12 h light/dark cycle and allowed full access to standard mouse chow and water.

The C57 mice were randomly divided into four groups: sham group, sham+Ang IV group, MI group, and MI+Ang IV group. MI was established by ligation of LAD, while surgery was performed without LAD ligation in the sham group. The sham+Ang IV and MI+Ang IV groups received continuous subcutaneous infusion of Ang IV (1.44 mg/kg/day) via an osmotic pump (model 2004; ALZET, CA, USA) 3 d before AMI.

The in vivo study was divided into two parts. In the first part, 20 mice underwent surgery and were raised for 30 d postoperatively. On the 30th postoperative day, the survival rate was assessed and echocardiography was performed. At the end of the experiment, all mice were sacrificed under anesthesia by an intraperitoneal injection of 0.8% pentobarbital sodium (60 mg/kg).

In the other part, at day 7 after surgery, 10 mice in each group were sacrificed with intraperitoneal injection of 0.8% pentobarbital sodium (60 mg/kg) and mouse hearts were removed for further analysis.

### 2.2. MI Model

LAD was ligated to induce MI in mice as previously described [16]. Briefly, all the operations were carried out under isoflurane (RWD Life Science, Shenzhen, China, 2%), and mice were ventilated with a small animal ventilator (Harvard Apparatus, MA, USA). Anesthesia was considered adequate when the pedal withdrawal reflex was negative. Then, the mouse hearts were exposed through the left lateral thoracotomy and MI was induced by permanent ligation of the LAD with a 7-0 suture line. Sham-operated mice underwent the same surgical procedure without LAD ligation. A warming blanket was used throughout the surgery.

### 2.3. Echocardiography

Transthoracic echocardiography was performed in mice before surgery and on day 30 after surgery on a Vevo 770 High-Resolution Imaging System (Visual Sonics, Canada) with a 35 MHz ultrasound probe as previously described [17]. Mice were anesthetized with isoflurane (2%). Left ventricular end-diastolic internal diameter (LVEDd), left ventricular end-systolic internal diameter (LVEDs), left ventricular fractional shortening (LVFS), and left ventricular ejection fraction (LVEF) were measured by the same observer.

### 2.4. Serum Biochemistry Assay

All mice were starved overnight, and blood samples were collected from the heart after sacrifice. Serum levels of total cholesterol (TC), triglycerides (TGs), low-density lipoprotein (LDL) cholesterol, and high-density lipoprotein (HDL) cholesterol were determined using an enzymatic assay with a biochemistry automatic analyzer (HITACHI 7170A, Hitachi, Tokyo, Japan).

### 2.5. Histology and Morphology

Euthanized mice were perfused with saline to eliminate blood in the lumen, and the hearts were removed and fixed in 4% paraformaldehyde (Beyotime, Nantong, China). Mouse hearts were embedded in an optimal cutting temperature (OCT) compound, and serial sections (5 *μ*m) were cut for hematoxylin and eosin (H&E) and Masson staining. Corresponding sections on separate slides were visualized under a microscope for morphological assessment.

### 2.6. Terminal Deoxyribonucleotidyl Transferase- (TDT-) Mediated dUTP Nick-End Labeling (TUNEL)

Apoptosis of mouse heart tissue was detected using the ApopTag Plus Peroxidase In Situ Apoptosis Detection Kit (Millipore, MA, USA) as previously described [18]. The apoptosis of neonatal rat ventricular myocytes (NRVCs) was determined using the in situ cell death detection kit (Roche, CA, USA). The number of TUNEL-positive cardiomyocyte nuclei and total number of cardiomyocyte nuclei in each site were counted. The ratio of apoptotic cardiomyocytes was calculated by dividing the number of TUNEL-positive cardiomyocyte nuclei by the total number of cardiomyocyte nuclei.

### 2.7. Transmission Electron Microscopy

Transmission electron microscopy was performed as described previously [19]. Briefly, the left ventricular tissues were sliced into ultrathin sections. These sections were fixed with 4% paraformaldehyde and 1% glutaraldehyde in phosphate-buffered saline (PBS) at 4°C overnight. The preparations were washed and dehydrated with increasing concentrations of ethanol, followed by embedding and sectioning. The cardiac slices were examined by microscopy under an H7650 transmission electron microscope (Hitachi, Tokyo, Japan).

### 2.8. Cell Culture and Stimulation

Primary ventricular cardiomyocytes were isolated from 1- to 2-day-old neonatal rat hearts [20]. NRVCs were cultured in Dulbecco's modified Eagle's medium (DMEM; ScienCell, CA, USA) supplemented with 10% fetal bovine serum, 100 U/ml penicillin, and 100 *μ*g/ml streptomycin at 37°C for 24 h. Cells were divided into four groups: the control group, hypoxia group, Ang IV group, and hypoxia+Ang IV group. The cells were exposed to hypoxic stimulation in a Whitley H35 hypoxystation (Don Whitley Scientific, London, England) under an atmosphere of 1% oxygen (O_2_), 5% carbon dioxide (CO_2_), and 94% nitrogen (N_2_) for 24 h [21]. The Ang IV and hypoxia+Ang IV groups were stimulated with Ang IV (1 *μ*M) for 24 h before hypoxia, while the control group was simulated with saline.

### 2.9. Real-Time Quantitative Polymerase Chain Reaction (PCR)

Total RNA was extracted from mouse hearts using the TRIzol reagent (Invitrogen, CA, USA). The mRNA expression levels of monocyte chemotactic protein 1 (MCP-1), intercellular adhesion molecule 1 (ICAM-1), and inducible nitric oxide synthase (iNOS) were determined using SYBR Green technology (Bio-Rad, CA, USA) with glyceraldehyde 3-phosphate dehydrogenase (GAPDH) as an internal control. Quantitative values were obtained from the threshold cycle value (Ct), and the 2^-△△Ct^ method was used to determine the relative gene expression levels. The primers were as follows: MCP-1, forward, 5′-TCTGGGCCTGCTGTTCACA-3′, reverse, 5′-GGATCATCTTGCTGGTGAATGA-3′; ICAM-1, forward, 5′-GCCTTGGTAGAGGTGACTGAG-3′, reverse, 5′-GACCGGAGCTGAAAAGTTGTA-3′; iNOS, forward, 5′-GTTCTCAGCCCAACAATACAAGA-3′, reverse, 5′-GTGGACGGGTCGATGTCAC-3′; and GAPDH, forward, 5′-AGGTCGGTGTGAACGGATTTG-3′, reverse, 5′-TGTAGACCATGTAGTTGAGGTCA-3′.

### 2.10. Western Blotting Analysis

Extracted proteins were quantified using a BCA Protein Assay Kit (Beyotime, Nantong, China). The protein extracts (50 *μ*g) were fractionated by sodium dodecyl sulfate-polyacrylamide gel electrophoresis (SDS-PAGE) and transferred to nitrocellulose membranes. After blocking with silk milk for 2 h at room temperature, the proteins were probed with primary antibodies and specific conjugated peroxidase-labeled secondary antibodies. The immunoreactive bands were visualized using a luminescent image analyzer (Amersham Imager 600; GE, MA, USA). Protein expression levels were determined by densitometry. All experiments were performed in triplicate. The primary antibodies used were as follows: rabbit anti-MCP-1 antibody (Cell Signaling Technology, MA, USA), rat ICAM-1 antibody (1 : 500; R&D Systems, MN, USA), rabbit anti-iNOS antibody (1 : 1000; Cell Signaling Technology), rabbit anti-LC3 antibody (1 : 1000; Cell Signaling Technology), rabbit anti-P62 antibody (1 : 1000; Cell Signaling Technology), and rabbit anti-GAPDH antibody (1 : 1000; Cell Signaling Technology).

### 2.11. Measurements of Reactive Oxygen Species (ROS)

ROS levels in NRVCs were determined using DHE (Beyotime, Nantong, China). NRVCs were plated in 96-well plates and treated with vehicle or Ang IV (1 *μ*M) before hypoxia. After the treatment, DHE was added and incubated for 30 min at 37°C and washed twice with warm PBS, and the fluorescence intensity was measured with a fluorescence confocal microscope (LSM 710; Carl Zeiss, Germany).

### 2.12. Comet Assay

The DNA damage of NRVCs was assessed using a comet assay kit (Trevigen, MD, USA), as previously described [22].

### 2.13. ELISA

Conditioned medium (CM) from cultured cells was collected and filtered to remove the cell debris. Cytokine (tumor necrosis factor- (TNF-) *α* and interleukin- (IL-) 1*β*) secretion in CM was assayed using an ELISA kit (RayBiotech, GA, USA), according to the manufacturer's instructions.

### 2.14. Statistical Analysis

Statistical analysis involved the use of SPSS v.16.0 software (SPSS Inc, Chicago, IL). Data are shown as the mean ± standard deviation (SD). Statistical evaluation was carried out by Student's *t-*test between two groups and by one-way analysis of variance (ANOVA) followed by post hoc analysis within multiple groups. Statistical significance was set at *P* < 0.05.

## 3. Results

### 3.1. Ang IV Improved the Survival Rate Post-MI

**Figure 1:**
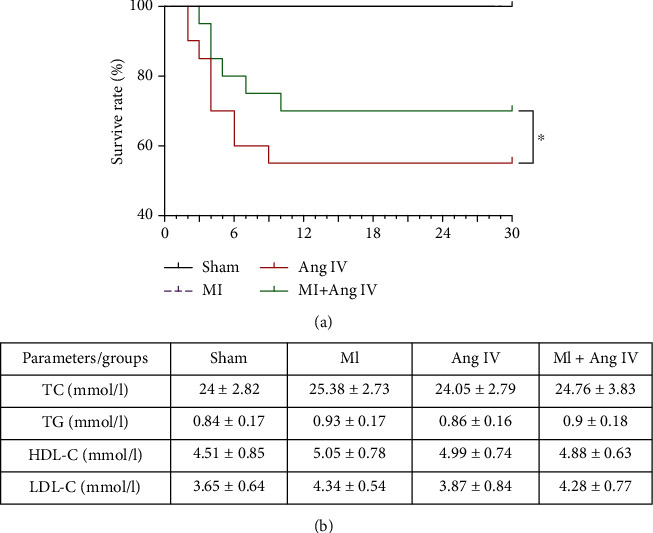
Ang IV increased survive rate after MI without any effect on lipid levels. (a) MI induced high mortality in mice, while Ang IV increased the survive rate after MI. (b) There was no significant differences in lipid levels in 4 groups of mice. D: days; TC: total cholesterol; TG: triglycerides; LDL: low-density lipoprotein cholesterol; HDL: high-density lipoprotein cholesterol; Ang: angiotensin; MI: myocardial infarction. ^∗^*P* < 0.05.

To examine the possible involvement of Ang IV in MI, we first compared the survival rates at day 30 after sham and MI operations. All mice in the sham and sham+Ang IV groups survived over the course of the 30-day study (Figure 1(a)). The incidence of mortality was remarkably lower in the MI+Ang IV group than in the MI group. The results indicated that Ang IV infusion played a protective role in MI. The plasma levels of TC, TGs, LDLs, and HDLs were analyzed. There was no significant difference among the four groups (Figure 1(b)), which suggested that the protective role of Ang IV was not associated with lipid levels.

### 3.2. Ang IV Infusion Reduced the Inflammatory Response after MI

**Figure 2:**
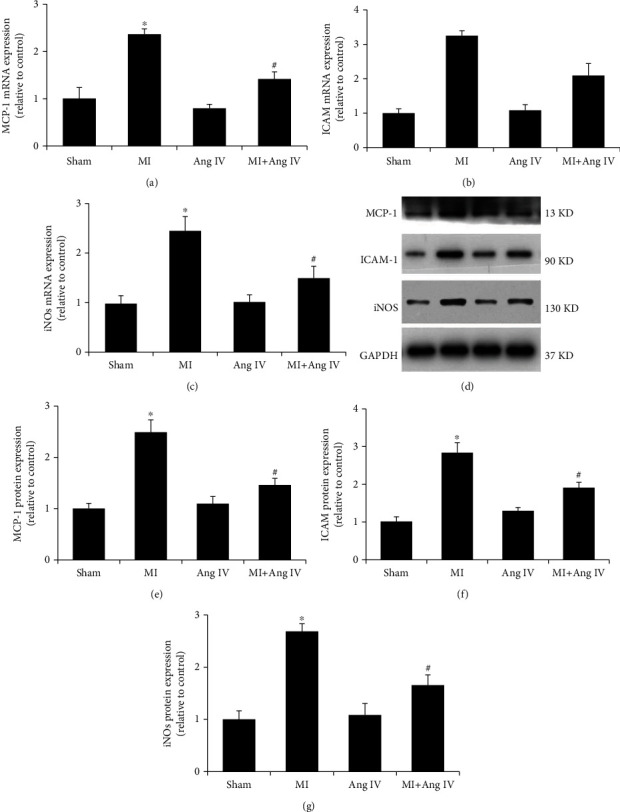
Ang IV reduced MI-induced inflammatory response in mice. (a–c) Ang IV reduced MI-induced mRNA expression of inflammatory cytokines, including MCP-1, ICAM-1, and iNOS. (d–g) Ang IV reduced MI-induced protein expression of inflammatory cytokines. MCP-1: monocyte chemotactic protein 1; ICAM-1: intercellular adhesion molecule 1; iNOS: inducible nitric oxide synthase; Ang: angiotensin; MI: myocardial infarction. ^∗^*P* < 0.05 vs. the sham group; ^#^*P* < 0.05 vs. the MI group.

The inflammatory response is a major contributor to ischemic cardiac injury, resulting from its significant impact on triggering myocardial apoptosis [23]. Inflammatory cytokines were measured in infarcted hearts 7 d after MI. The mRNA and protein expression levels of MCP-1, ICAM-1, and iNOS were consistently and markedly elevated in the MI hearts compared with the sham group (Figure 2) but significantly attenuated in the heart tissue of the MI+Ang IV group. These results suggest that Ang IV exerts an anti-inflammatory effect after MI.

### 3.3. Ang IV Infusion Reduced Apoptosis and Autophagosomes after MI

**Figure 3:**
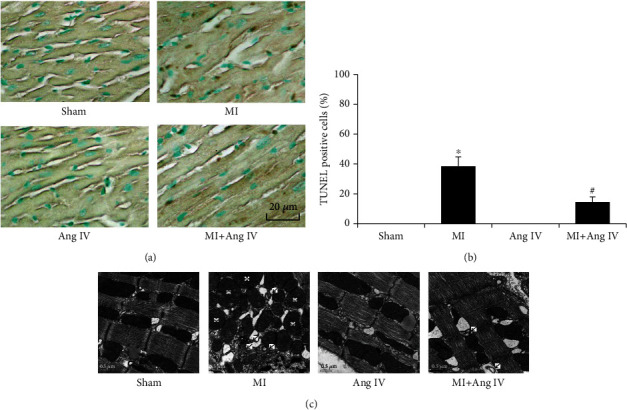
Ang IV reduced apoptosis and autophagosomes in MI mice heart. (a, b) TUNEL staining showed that Ang IV reduced MI-induced cardiomyocyte apoptosis. Bar = 20 *μ*m. (c) Transmission electron microscopy showed that MI induced autophagosomes and mitochondrial swelling and disarrangement, while Ang IV infusion could improve it. Bar = 0.5 *μ*m. Ang: angiotensin; MI: myocardial infarction. ^∗^*P* < 0.05 vs. the sham group; ^#^*P* < 0.05 vs. the MI group. Arrow, autophagosomes; cross, mitochondrial.

We then examined whether there was any difference in cardiomyocyte apoptosis in the border zone. TUNEL assay results showed that in the border zone, there was no cardiomyocyte apoptosis in the sham and Ang IV groups; however, MI significantly induced cardiomyocyte apoptosis and Ang IV infusion markedly reduced the percentage of TUNEL-positive cells in the MI+Ang IV group (Figures 3(a) and 3(b)). Transmission electron microscopy showed that compared with MI mice, Ang IV reduced autophagosomes in cardiomyocytes and improved mitochondrial swelling and disarrangement (Figure 3(c)).

### 3.4. Ang IV Infusion Improved Cardiac Remodeling after MI

**Figure 4:**
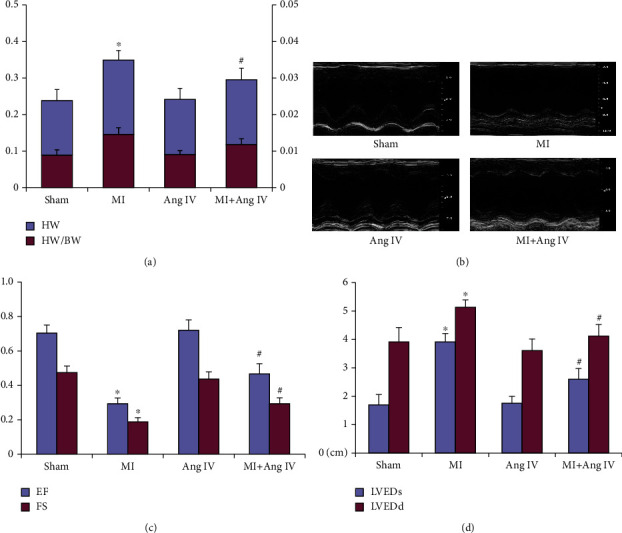
Ang IV improved cardiac function after MI in mice. (a) MI increased the HW and the ratio of HW/BW, while infusion of Ang IV reduced them. (b–d) Echocardiography showed that Ang IV improved cardiac function after MI, including EF, FS, LVEDs, and LVEDd. Ang: angiotensin; MI: myocardial infarction; HW: heart weight (g); BW: body weight (g); EF: ejection fraction; FS: fractional shortening; LVEDs: left ventricular end systolic internal diameter; LVEDd: left ventricular end diastolic internal diameter. ^∗^*P* < 0.05 vs. the sham group; ^#^*P* < 0.05 vs. the MI group.

The ratio of heart weight to body weight was measured in the following experiments. As shown in Figure 4(a), 30 d after MI, the ratio was higher in the MI group than in the sham group, while Ang IV infusion reduced the ratio in the MI+Ang IV group. In addition, there was no difference in baseline cardiac function and geometry among the four groups of mice (data not shown). However, at day 30 after MI, a greater deterioration in LV function was observed in the MI group, while Ang IV improved post-MI cardiac dysfunction, including LVEDs, LVEDd, FS, and EF (Figures 4(b)–4(d)). The results indicated that Ang IV treatment improved heart remodeling after MI.

### 3.5. Ang IV Infusion Reduced Infarct Size and Fibrosis after MI

**Figure 5:**
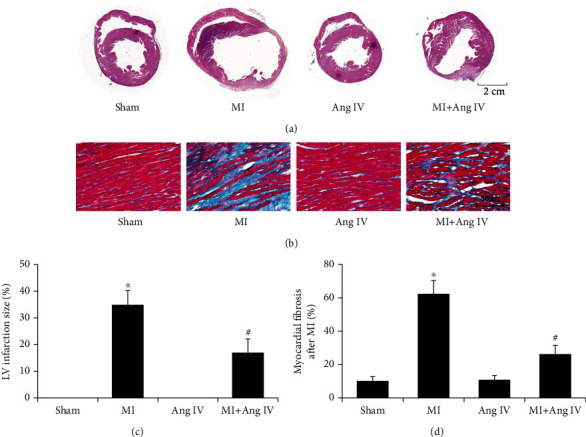
Ang IV reduced infarct size and fibrosis after MI in mice. (a, c) H&E staining showed that Ang IV infusion reduced MI-induced infarct size. Bar = 2 cm; (b, d) Masson staining showed that Ang IV infusion reduced MI-induced cardiac fibrosis. Bar = 50 *μ*m. Ang: angiotensin; MI: myocardial infarction; ^∗^*P* < 0.05 vs. the sham group; ^#^*P* < 0.05 vs. the MI group.

In the following experiment, cardiac remodeling was analyzed 30 d after MI. H&E staining revealed that there were similar morphologies in the mice of the sham and sham+Ang IV groups, whereas MI mice demonstrated heart expansion and myocardial infarction with a large area, while Ang IV infusion significantly reduced the infarct area and heart expansion (Figures 5(a) and 5(c)). Moreover, Masson staining showed that MI induced fibrosis in the infarcted hearts, while Ang IV reduced fibrosis in the MI+Ang IV mice (Figures 5(b) and 5(d)).

### 3.6. Ang IV Reduced Hypoxia-Induced Oxidative Stress and DNA Damage *In Vitro*

**Figure 6:**
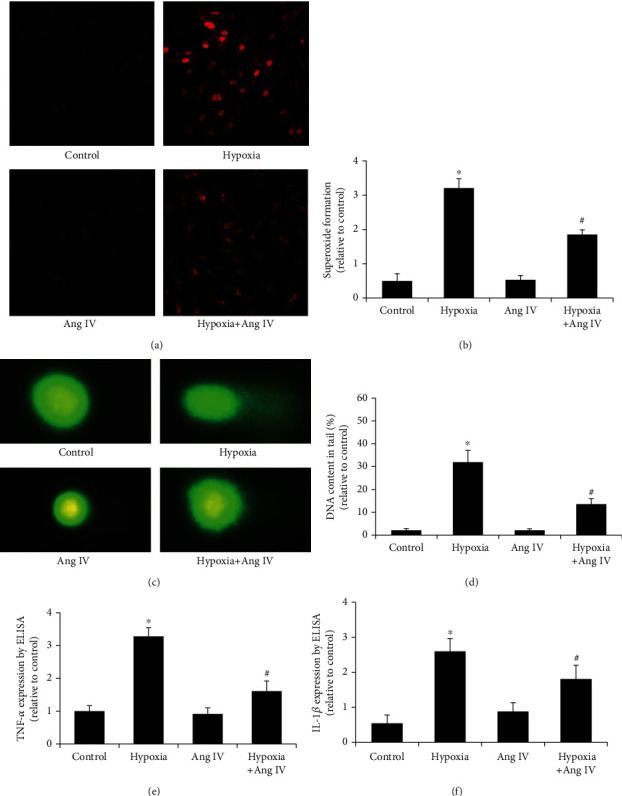
Ang IV reduced hypoxia-induced oxidative stress and DNA damage as well as inflammatory cytokine secretion in NRVCs. (a, b) DHE staining showed that pretreatment of Ang IV reduced oxidative stress. (c, d) Comet assay showed that Ang IV reduced hypoxia-induced DNA damage. (e, f) ELISA showed that Ang IV reduced hypoxia-induced TNF-ɑ and IL-1*β* secretion in supernatant. Ang: angiotensin; TNF-ɑ: tumor necrosis factor ɑ; IL-1*β*: interleukin 1*β*; NRVCs: the neonatal rat ventricular myocytes. ^∗^*P* < 0.05 vs. the control group; ^#^*P* < 0.05 vs. the hypoxia group.

NRVCs were first stimulated by Ang IV before hypoxia, and oxidative stress was analyzed by DHE staining. Compared with the control, hypoxia significantly induced oxidative stress in NRVCs, while Ang IV markedly reduced it (Figures 6(a) and 6(b)). Moreover, comet assay showed that there was little DNA in the nuclear tail, whereas hypoxia increased the DNA content in the tail, and pretreatment with Ang IV dramatically reduced it (Figures 6(c) and 6(d)). The levels of TNF-*α* and IL-1*β* in the supernatant were analyzed by ELISA. Hypoxia stimulation increased the secretion of TNF-*α* and IL-1*β*, while pretreatment with Ang IV reduced it (Figures 6(e) and 6(f)).

### 3.7. Ang IV Reduced Cardiomyocyte Apoptosis and Autophagy Induced by Hypoxia *In Vitro*

**Figure 7:**
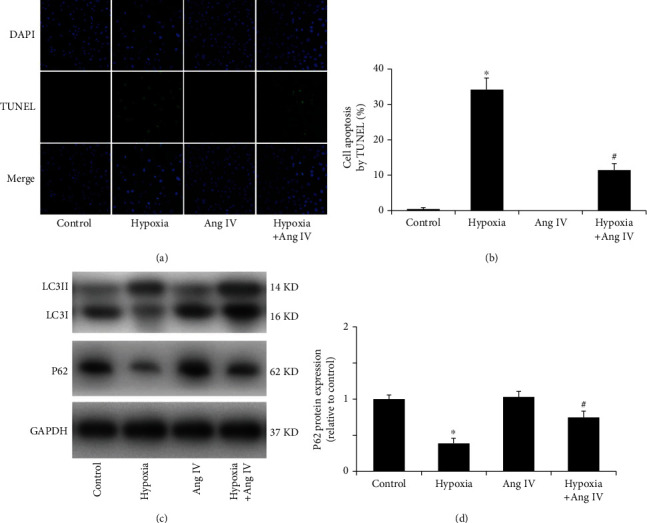
Ang IV reduced hypoxia-induced apoptosis and autophagy in NRVCs. (a, b) TUNEL staining showed that Ang IV reduced hypoxia-induced cardiomyocyte apoptosis. Bar = 100 *μ*m. (c, d) Western blot analysis showed that Ang IV reduced the transformation of LC3-I to LC3-II but increased p62 expression. Ang: angiotensin; NRVCs: the neonatal rat ventricular myocytes. DAPI: blue; TUNEL: green; ^∗^*P* < 0.05 vs. the control group; ^#^*P* < 0.05 vs. the hypoxia group.

Apoptosis of cardiomyocytes was analyzed by TUNEL assay. Immunofluorescence showed that there was little cell apoptosis in the control and Ang IV groups, and hypoxia significantly increased the number of apoptotic cardiomyocytes, whereas pretreatment with Ang IV reduced apoptosis (Figures 7(a) and 7(b)). To further investigate the probable mechanism, we evaluated the protein levels of LC3 and p62, which are indicators of autophagy. Western blotting analysis revealed that the expression of p62 was reduced in hypoxic cells, while Ang IV increased the expression of p62. Moreover, compared with the hypoxia group, Ang IV reduced the transformation of LC3-I to LC3-II. Taken together, our study showed that Ang IV protected against hypoxia-induced apoptosis by inhibiting autophagy *in vitro*.

## 4. Discussion

AMI remains a major cause of morbidity and mortality worldwide [24]. With the development in medicine, especially percutaneous coronary intervention (PCI), the mortality of AMI has dramatically decreased [25]. However, heart failure and remodeling after AMI are serious issues that significantly reduce the quality of life [26]. Thus, the development of novel and effective pharmacological treatments is urgently needed. This study characterized Ang IV as a novel treatment for MI. Continuous infusion of Ang IV can reduce inflammation, apoptosis, and fibrosis, resulting in reduced infarct size, improved cardiac function, and survival *in vivo*. Furthermore, Ang IV reduced hypoxia-induced oxidative stress and DNA damage in vitro. Ang IV reduces apoptosis by inhibiting autophagy. To the best of our knowledge, these results provide the first direct evidence that Ang IV preserves cardiac dysfunction after MI and has important clinical implications for the treatment of ischemic cardiac injury.

Angiotensin [3–8] (Ang) IV, a hexapeptide fragment of Ang II formed from Ang III via the action of aminopeptidase N, binds with high affinity to the designated AT4 receptor (AT4R) [27]. Chronic Ang IV treatment reverses endothelial dysfunction in apolipoprotein E- (ApoE-) deficient mice [14]. In addition, Ang IV has a cardioprotective effect against ischemia-reperfusion (I/R) injury by inhibiting apoptosis via the AT4R and the phosphoinositide 3-kinase/serine-threonine kinase/mammalian target of rapamycin (PI3K/Akt/mTOR) pathway [28]. However, angiotensin peptides have a very short half-life [15]. Thus, only a constant infusion of Ang IV can maintain a high serum Ang IV concentration and exhibit some effects. In a previous study, Ang IV was continuously infused by a minipump, which was also used in our experiment [15]. We found that continuous infusion of Ang IV increased survival after MI, and the therapeutic effect of Ang IV was independent of lipid profiles.

Although postinfarction inflammation is important for the removal of necrotic cardiomyocytes and extracellular matrix debris [29], prolonged inflammation directly promotes myocardial apoptosis and exerts a detrimental effect on the pathological process of MI [30]. Kong et al. found that Ang IV treatment markedly reduced macrophage infiltration and proinflammatory cytokines [15], suggesting its anti-inflammatory effect. In our study, MI increased the expression levels of inflammatory cytokines, including MCP-1, ICAM-1, and iNOS; however, continuous infusion of Ang IV reduced the mRNA and protein expression levels of these inflammatory cytokines. *In vitro*, hypoxia induced ROS production and DNA damage, which could be alleviated by Ang IV. Moreover, pretreatment with Ang IV significantly reduced the secretion of TNF-*α* and IL-1*β*. The *in vivo* and *in vitro* results demonstrated that Ang IV had an anti-inflammatory effect. However, further experiments are required to elucidate the cell signal transduction mechanisms.

Apart from inflammation in MI, cardiac fibrosis is also an important pathological process that contributes to the pathogenesis of cardiac remodeling after MI, which is a transition from an early inflammatory phase to fibrotic granulation and the maturation stage of cardiac remodeling [16]. Myocardial fibrosis is the end point of cell differentiation and the activation and proliferation of cardiac fibroblasts [31, 32]. Inhibition of fibrosis is a promising therapy for cardiac remodeling after MI. The angiotensin receptor neprilysin inhibitor, LCZ696, attenuates cardiac remodeling and dysfunction after MI by reducing cardiac fibrosis and hypertrophy [33]. Our study is the first to demonstrate that continuous infusion of Ang IV can alleviate fibrosis in the infarct area after MI.

As the regenerative ability of the myocardium is very limited, apoptosis essentially contributes to cardiomyocyte loss after MI, which is directly responsible for increased infarct size in ischemic heart and the subsequent cardiac dysfunction or even heart failure [34]. Thus, effective attenuation of apoptosis can reduce the infarct size after MI [35]. It is also the reason we assessed the effect of Ang IV on cardiomyocyte apoptosis in our study. Ang IV was found to play a previously unrecognized role in the protection against myocardial apoptosis, with a reduced number of apoptotic cardiomyocytes in the mouse heart and cardiomyocytes. In fact, apoptosis-induced cardiomyocyte loss is a common pathological process shared by many cardiomyopathy-related disorders [36]. Thus, our study may provide clues regarding the role of Ang IV in other types of apoptosis-related cardiomyopathies, such as dilated cardiomyopathy and peripartum cardiomyopathy. The interruption of homeostasis between pro- and antiapoptotic proteins is the underlying cause of myocardial apoptosis after MI [37]. In this study, we investigated the probable mechanism for this. Cardiomyocyte apoptosis and autophagy are fundamental for cardiac homeostasis, repair, and remodeling [38]. Autophagy is induced by various stressors and maintains an optimal cellular environment by removing protein aggregates and damaged organelles [39]. Autophagy is a complex process that involves multiple factors. In mammals, LC3B-I is conjugated with phosphatidylethanolamine to form LC3B-II, resulting in the maturation of autophagosomes [40]. Dysregulation of autophagy is associated with a number of cardiac diseases, including cardiomyopathy [41], ischemic heart disease [42], and heart failure [43]. There are some controversial studies regarding the role of autophagy and the effect of the interaction between autophagy and apoptosis in cell survival during MI [39]. In our experiment, autophagy was overactivated by MI, but Ang IV inhibited it both *in vivo* and *in vitro*. Mitochondrial dysfunction plays a critical role in heart failure after MI [44], which was also investigated in the present study. We found that MI induced the swelling and disarrangement of mitochondria in cardiomyocytes, whereas Ang IV infusion improved this abnormality.

There were some limitations to this experiment. First, it was very difficult to obtain human tissue to carry out human tests; second, the precise mechanism or signaling pathway involved could not be clearly identified, which needs to be further explored in future experiments.

In conclusion, these data suggest that long-term infusion of Ang IV before and after MI improves the prognosis and attenuates chronic post-MI cardiac remodeling by inhibiting the inflammatory response and reducing apoptosis via the inhibition of autophagy. Therefore, Ang IV may be a promising treatment for improving the prognosis of post-MI cardiac remodeling in patients with reduced LVEF after AMI.

## Data Availability

The data was available under reasonable request from the corresponding author.
