# Correction to “Continuous Infusion of Angiotensin IV Protects against Acute Myocardial Infarction via the Inhibition of Inflammation and Autophagy”

**DOI:** 10.1155/omcl/9781730

**Published:** 2025-10-21

W. Bai, H. Wang, C. Gao, et al., “Continuous Infusion of Angiotensin IV Protects against Acute Myocardial Infarction via the Inhibition of Inflammation and Autophagy,” *Oxidative Medicine and Cellular Longevity* 2021 (2021): 2860488, https://doi.org/10.1155/2021/2860488.

In the article titled, “Continuous Infusion of Angiotensin IV Protects against Acute Myocardial Infarction via the Inhibition of Inflammation and Autophagy,” there was an error in Figure 1a. In Figure 1 legend, Ang IV and MI were attributed to the wrong color. The corrected figure is shown below and is listed as Figure 1:

We apologize for this error.

## Figures and Tables

**Figure 1:**
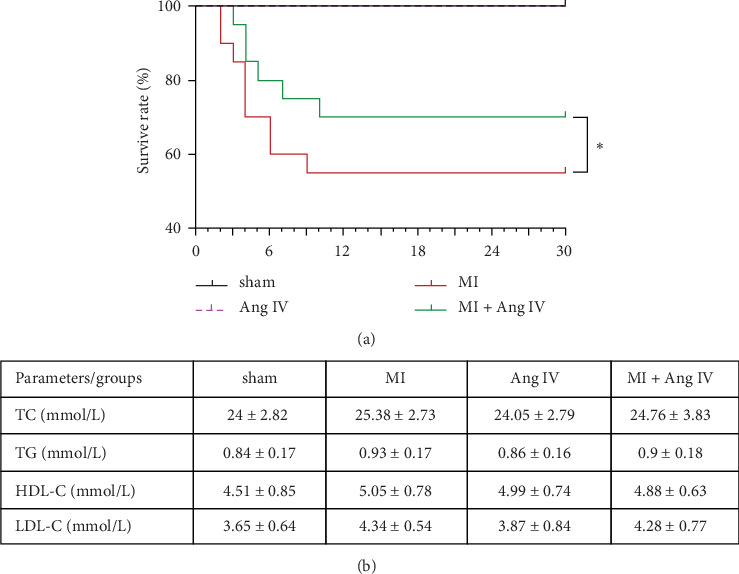
Ang IV increased survive rate after MI without any effect on lipid levels. (a) MI induced high mortality in mice, while Ang IV increased the survive rate after MI. (b) There was no significant differences in lipid levels in four groups of mice. Ang, angiotensin; D, days; HDL, high-density lipoprotein cholesterol; LDL, low-density lipoprotein cholesterol; MI, myocardial infarction; TC, total cholesterol; TG, triglycerides. *⁣*^*∗*^*p* < 0.05.

